# Lupusnephritis und assoziierte thrombotische Mikroangiopathie

**DOI:** 10.1007/s00393-024-01489-9

**Published:** 2024-03-05

**Authors:** Antonia Schuster, Bernhard Banas, Tobias Bergler

**Affiliations:** 1 Abteilung für Nephrologie, Universitätsklinikum Regensburg, Franz-Josef-Strauß-Allee 11, 93053 Regensburg, Deutschland; 2 Medizinische Klinik III-Nephrologie, Klinikum Ingolstadt, Ingolstadt, Deutschland

**Keywords:** Lupusnephritis, Thrombotische Mikroangiopathie, Plasmaaustausch, Komplementinhibition, Eculizumab, Lupus nephritis, Thrombotic microangiopathy, Plasma exchange, Complement inhibition, Eculizumab

## Abstract

Die Lupusnephritis stellt die häufigste Manifestation eines systemischen Lupus an den soliden Organen dar und geht mit einem erhöhten Risiko für eine chronische Niereninsuffizienz einher. Das gleichzeitige Auftreten einer Lupusnephritis mit einer thrombotischen Mikroangiopathie wird als selten beschrieben, impliziert jedoch das Risiko fataler Organdysfunktionen. Wir berichten von drei Patienten, bei denen diese beiden Krankheitsentitäten parallel auftraten und eine intensivierte immunsuppressive Therapie auch mittels Komplementblockade notwendig machten.

## Abstract (en)

Lupus nephritis represents the most common manifestation of lupus of the solid organs and is associated with an increased risk of chronic kidney disease. The co-occurrence of lupus nephritis and thrombotic microangiopathy is described to be rare but implies the risk of fatal organ dysfunction. We report three patients in whom these two disease entities occurred in parallel, necessitating intensive immunosuppressive therapy, including complement blockade.

## Einleitung

Der systemische Lupus erythematodes (SLE) ist eine multifaktoriell bedingte Systemerkrankung, bei der es zu entzündlichen Gefäß- und Bindegewebeveränderungen und Immunkomplexablagerungen kommt. Der Erkrankungsgipfel liegt zwischen dem 20. und 30. Lebensjahr, wobei Frauen etwa 9 × häufiger betroffen sind. Neben der Haut können alle Organe betroffen sein. Die Niere (Lupusnephritis, LN) ist der häufigste Manifestationsort der soliden Organe und geht mit einem erhöhten Risiko für die Entwicklung einer chronischen Niereninsuffizienz sowie einer erhöhten Morbidität und Mortalität einher [1, 2].

Die thrombotische Mikroangiopathie (TMA) umfasst eine Reihe unterschiedlicher Krankheitsentitäten, welche durch die Bildung von Mikrothromben in den Arteriolen/Kapillaren mit Mikrozirkulationsstörung und Endothelschäden charakterisiert sind, und bei einer hämolytischen Anämie, schweren Thrombozytopenie und ggf. bedrohlichen Organdysfunktionen einer sofortigen Therapieeinleitung bedürfen [3, 4].

In der Literatur finden sich vereinzelte Fallberichte mit dem Nachweis histologischer, TMA-typischer Veränderungen und serologischer Zeichen einer thrombotisch-thrombozytopenischen Purpura (TTP) oder eines atypischen hämolytischen Syndroms (aHUS) parallel zur Diagnose einer Lupusnephritis. Eine Aussage inwieweit diese Krankheitsentitäten sich gegenseitig bedingen bzw. inwieweit das Outcome der Lupusnephritis durch das Auftreten einer TMA verändert wird, ist jedoch derzeit noch unklar [5–7].

Im Folgenden möchten wir über drei Patienten berichten, die durch das simultane Auftreten einer Lupusnephritis und einer TMA auffielen.

## Fallberichte

Der *erste Patient*, 29 Jahre, wurde extern mit respiratorischer Hypoxämie nach grippalem Infekt behandelt. Zusätzlich fielen bds. Pleuraergüsse, ein hämodynamisch relevanter Perikarderguss und eine hochgradig reduzierte linksventrikuläre Pumpfunktion auf, weswegen u. a. eine Perikarddrainage angelegt wurde. Laborchemisch zeigten sich ein dialysepflichtiges Nierenversagen mit Erythrozyturie und Albuminurie sowie eine transfusionspflichtige Anämie und Thrombopenie. Es zeigten sich ein erhöhter ANA(Antinukleäre Antikörper)- und Anti-Doppelstrang-DNA-Titer (Immunoassay) sowie ein Komplementverbrauch. Die Anti-Phospholipid-Antikörperbestimmung verblieb negativ. Aufgrund o. g. Auffälligkeiten wurde bei Erstdiagnose eines SLE mit assoziierter LN vom „Rapid-progressive-Glomerulonephritistyp“ eine Prednisolon-Stoßtherapie eingeleitet. Die durchgeführte Nierenbiopsie ergab den Befund einer Lupusnephritis der Klasse IV nach ISN/RPS, weswegen die Therapie um Cyclophosphamid erweitert wurde.

Serologisch zeigte sich additiv eine hämolytische Anämie (erhöhtes LDH, Nachweis von Fragmentozyten, nicht messbares Haptoglobin) als Hinweis auf eine TMA, die sich auch histologisch in der Nierenbiopsie nachvollziehen ließ. Es konnten in 3 Arteriolen frische TMA-artige Veränderungen nachgewiesen werden. Es wurde zur Akutintervention ein Plasmaaustauschverfahren eingeleitet. Nachdem es unter Pausierung des selbigen zu einem Rebound der o. g. Hämolyseparameter kam, wurde eine Komplementblockade mit Eculizumab initiiert. Hierunter zeigten sich die Nierenfunktionsparameter gebessert, die Dialysetherapie konnte beendet werden. Insgesamt erhielt der Patient 7 Plasmapheresen à 19 FFPs, Eculizumab nach Standard mit einer Erhaltungstherapie (4 Monate) und Cyclophosphamid (kumulativ 2910 mg). Die Erhaltungstherapie wurde mit Mycophenolat Mofetil (MMF) initiiert. Aktuell zeigen sich die Nierenfunktion und die Lupusparameter stabil.

Die *zweite Patientin*, 37 Jahre, wurde durch den Hausarzt mit akutem Nierenversagen, Thrombopenie und Anämie eingewiesen. Anamnestisch gab die Patientin Husten, Schnupfen sowie eine Raynaud-Symptomatik, Arthralgien und Dyspnoe an. Es zeigte sich eine Albuminurie und in der Urinmikroskopie ein aktives nephritisches Sediment. Serologisch zeigten sich erhöhte ANA- und Anti-dsDNA-Titer und ein Komplementverbrauch. Die Anti-Phospholipid-Antikörper fielen negativ aus. Es wurde die Diagnose eines Lupus erythematodes gestellt und ein Prednisolonstoß initiiert. Dieser wurde im Verlauf oralisiert (1 mg/kgKG) und zeitnah eine Therapie mit MMF gestartet. In der ergänzten Nierenbiopsie erfolgte der Nachweis einer Lupusnephritis der Klasse III nach IRS/RPS mit mäßigen Aktivitäts- und Chronizitätszeichen. Zwei Wochen nach der Entlassung stellte sich die Patientin nochmals selbstständig aufgrund einer deutlichen Gewichtszunahme von 20 kg, Dyspnoe und Diarrhoen vor. Laborchemisch zeigten sich die Nierenretentionsparameter inklusive der Albuminurie stabil zu den Vorbefunden. Es konnten aber erneut eine verstärkte Thrombopenie und eine Anämie sowie Hämolysezeichen mit einem Haptoglobinverbrauch, erhöhter LDH und Fragmentozyten im Blutausstrich nachgewiesen werden. Nachdem sowohl die serologischen Lupusaktivitätsparameter als auch die renalen Parameter stabil waren, wurde in Zusammenschau der V. a. eine sekundäre thrombotische Mikroangiopathie gestellt. Ein erneuter Schub des SLE mit autoimmunhämolytischer Anämie konnte aufgrund der stabilen Laborwerte und nachweislichen Fragmentozyten ausgeschlossen werden. Von einer erneuten Nierenbiopsie wurde auch in Hinblick auf die Thrombopenie Abstand genommen. Es wurde eine Komplementblockade mit Eculizumab, (4 × 900 mg) begonnen. Unter dieser Therapie normalisierten sich die Hämolyseparameter. In der Nachsorge stellten sich das Blutbild, die Nierenretentionswerte und Albuminurie gebessert dar ohne Hinweise auf anhaltende Lupusaktivität.

Die Erstdiagnose des SLE erfolgte bei der *dritten Patientin* (30 Jahre) einige Jahre zuvor mit Myalgien, Arthralgien, einer Serositis und Panzytopenie sowie positiven ANA- und Anti-Doppelstrang-DNA-Titern. Eine damalige Nierenbiopsie ergab den Befund einer Lupusnephritis der Klasse IV nach IRS/RPS. Eine Medikation mit Prednisolon, MMF und Hydroxychloroquin wurde initiiert. Sechs Jahre später stellte sich die Patientin notfallmäßig wegen eines akuten, anuren Nierenversagens, hypertensiven Blutdruckwerten sowie einer Anämie und Thrombopenie vor. Die Anti-Phospholipid-Antikörper fielen negativ aus. Klinisch konnte eine Serositis mit Perikarderguss, Pleuraerguss und Aszites nachgewiesen werden. Somit erfolgte bei Schub des SLE die Einleitung einer Therapie mit hoch dosiertem Prednisolon und Cyclophosphamid i.v. Zudem zeigten sich neben der Thrombopenie und Anämie Hämolysezeichen mit einem Haptoglobinverbrauch, einer erhöhten LDH sowie Fragmentozyten im Blutausstrich, sodass die Diagnose einer additiven thrombotischen Mikroangiopathie gestellt und zusätzlich mit Plasmaseparation gestartet wurde. Nachdem unter Pausierung der Plasmapherese zügig ein erneuter Anstieg der Hämolyse zu verzeichnen war, wurde bei Annahme eines komplement-assoziierten HUS die Entscheidung zur Therapie mit Eculizumab getroffen. Von einer Nierenbiopsie wurde aufgrund der Laborkonstellation (protrahierte Hämolyse mit Nachweis von Fragmentozyten) und dem deutlich reduzierten Allgemeinzustand der Patientin Abstand genommen. Trotz intensiver Therapie entwickelte die Patientin einen generalisierten Krampfanfall, der die Intubation erforderlich machte. Im MRT des Schädels präsentierte sich das Bild einer PRESDDZNS-Mitbeteiligung bei SLE. Unter Fortführung der o. g. Therapien, ausgeweiteter antihypertensiver Medikation und Negativbilanzierung durch die Dialyse besserte sich der Zustand. Auch die Lupusaktivitätsparameter und Hämolysezeichen zeigten sich im Verlauf konsolidiert. Die Erhaltungstherapie wurde mit MMF, Hydroxychloroquin und Prednisolon durchgeführt. Hierunter werden auch 2 Jahre später stabile serologische Aktivitätsparameter und eine gute Nierenfunktion festgestellt.

Tab. 1 zeigt den Verlauf der Laborparameter der 3 Patienten.

**Tab. 1:** Laborparameter über den Beobachtungszeitraum von 12 Monaten

		Aufnahme	Entlassung	3 Monate	6 Monate	12 Monate
*PAT 1*	Kreatinin (mg/dl)	6,55	2,12	1,92	1,45	1,27
	eGFR (ml/min)	11	42	47	66	78
	LDH (U/l)	454	489	262	166	169
	ACR (mg/g Krea)	1349	5847	624	417	231
	PCR (mg/g Krea)	1768	6941	748	458	323
	dsDNA (IU/ml)	2851,1	191,8	100,4	81,1	67,5
	C3 (mg/dl)	37,3	74,8	90,6	90,2	96,7
	C4 (mg/dl)	5,1	15,9	20,1	22,3	24,4
	Hb (g/dl)	8,8	7,6	10,4	10,2	13,8
	Thrombozyten (nl)	55	92	168	204	172
*PAT 2*	Kreatinin (mg/dl)	2,78	3,07	2,09	1,63	1,28
	eGFR (ml/min)	21	19	29	40	53
	LDH (U/l)	338	320	171	152	186
	ACR (mg/g Krea)	3800	1894	879	224	456
	PCR (mg/g Krea)	5554	2780	1147	324	569
	dsDNA (IU/ml)	1174,5	498,7	27,3	41,7	42,5
	C3 (mg/dl)	33,6	31,4	70	86,3	97,6
	C4 (mg/dl)	4,1	4,9	21,1	26,9	23,2
	Hb (g/dl)	7,0	7,8	8,6	9,5	12,8
	Thrombozyten (nl)	70	205	249	282	249
*PAT 3*	Kreatinin (mg/dl)	5,14	2,4	1,9	1,52	1,17
	eGFR (ml/min)	11	26	35	46	62
	LDH (U/l)	581	353	231	162	154
	ACR (mg/g Krea)	6569	4848	1766	3195	505
	PCR (mg/g Krea)	7803	5532	2130	3338	603
	dsDNA (IU/ml)	1344,0	192,3	210,7	163,7	124,5
	C3 (mg/dl)	24,8	65,6	78,5	93,9	105
	C4 (mg/dl)	2	9,8	13,7	15,1	24
	Hb (g/dl)	6,2	8,7	8,9	9,5	8,7
	Thrombozyten (nl)	60	47	192	214	241

## Diskussion

Eine Mitbeteiligung der Niere beim systemischen Lupus erythematodes ist häufig (1/3). Das gleichzeitige Auftreten eines SLE mit einer TMA wird in der Literatur als sehr selten beschrieben [5]. Wir können von drei Patienten berichten, bei denen es parallel zu einem Schub eines SLE und zu einer TMA mit Organdysfunktion gekommen ist.

Einer thrombotischen Mikroangiopathie können ätiologisch verschiedene Ursachen zugrunde liegen. Bei laborchemischem V. a. einer TMA ist daher die Initiierung einer schnellen Labordiagnostik zur differentialdiagnostischen Abklärung (ADAMTS-13-Aktivität, Shigatoxin, Komplementdiagnostik etc.) unabdingbar. Bis die Diagnostik vollständig vorliegt und eine valide Diagnose gestellt werden kann, sollte der Plasmaaustausch als „Bridging-Verfahren“ zur Stabilisierung des Patienten, v. a. bei schweren Fällen erwogen werden. Die Stuhldiagnostik wurde bei allen Patienten durchgeführt und ergab ein unauffälliges Ergebnis. Auch die Anti-Phospholipid-Antikörper wurden bestimmt und zeigten ebenso wie die ADAMTS-13-Aktivität keine Auffälligkeiten bei den Patienten, sodass die in Betracht kommenden Differentialdiagnosen bei den drei Patienten ausgeschlossen werden konnten. Nach Ausschluss der anderen Differentialdiagnosen und bei Vorliegen einer Komplementaktivierung (sC5b-9 = MAC) konnte daher die Diagnose eines aHUS gesichert werden. Eine Therapie mit dem monoklonalen Antikörper Eculizumab zur Komplementinhibition, welcher den Faktor C5 hemmt, ist daher indiziert und stellt inzwischen bei einer aHUS-Erkrankung die Erstlinientherapie dar, insofern die relevanten Differentialdiagnosen, welche einen Plasmaaustausch zwingend notwendig machen würden, zeitnah ausgeschlossen werden konnten. Daher wurde die entsprechende Therapie nach Diagnoseetablierung bei unseren Patienten auch eingeleitet. Erst hierdurch konnte ein klinisches Ansprechen beobachtet werden, was die Diagnose einer sekundären TMA stützt. Der Nachweis einer entsprechenden genetischen Mutation ist zur Diagnosestellung nicht zwingend erforderlich, sollte im Verlauf mittels humangenetischer Untersuchung aber angestrebt werden. Veränderungen in der Histologie können, müssen aber nicht immer nachvollzogen werden und geben keinen Anhalt auf die genau zugrunde liegende Ursache. Bei unserem ersten Patienten konnten in der Histologie in 3 Arteriolen frische TMA-artige Veränderungen nachvollzogen werden, was die Diagnose unterstreicht. Ein allgemeiner Konsens bezüglich der richtigen Therapiedauer von Eculizumab liegt bisher jedoch nicht vor, sodass es weiterhin eine patientenindividuelle Entscheidung unter Berücksichtigung der vorliegenden genetischen Mutationen, des bisherigen Erkrankungsverlaufs und Schweregrads, der Familienanamnese und der Nierenfunktion bleibt [8].

Diese Beobachtung bestätigt sich in der Literatur. Kim et al. berichteten von einem Patienten, der zu seinem Lupus ein durch eine C3-Mutation bedingtes aHUS entwickelte. Auch hier war der Einsatz von Eculizumab notwendig [6]. Ähnliche Ergebnisse konnten sowohl Park in ihrer Arbeit als auch Torres bei einer schwangeren Patientin nachweisen [9, 10].

Es stellt sich die Frage, inwieweit das gleichzeitige Auftreten dieser beiden Erkrankungsbilder das renale Outcome beeinflusst. Durch die erweiterte Therapie konnten wir bei unseren Patienten auch im mittelfristigen Verlauf eine Verbesserung der Nierenfunktion erzielen. In der Literatur finden sich kontroverse Aussagen. Chen et al. untersuchten 42 Patienten mit Lupus und TMA und verglichen diese mit 85 Patienten ohne additive TMA. Hier konnte gezeigt werden, dass Patienten mit TMA durch schwerwiegendere klinische Symptome und ein verschlechtertes 3‑Jahres-Gesamt- und renales Überleben auffielen [5]. Dahingegen konnte Massicotte-Azarniouch zwar eine schwere klinische Symptomatik bei den Patienten beobachten, jedoch ohne Unterschied in der Nierenfunktion. Einschränkend ist hier zu sagen, dass sich die Analyse auf den histologischen Befund einer TMA bezieht [7]. In Zusammenschau der Literatur muss angemerkt werden, dass sich die Daten, wie auch unsere, auf Fallberichte mit kleinen Fallzahlen beziehen. Eine klare Beurteilung des tatsächlichen Risikos und eine allgemeingültige Therapieempfehlung sind somit nur schwer möglich. Es lässt sich schlussfolgern, dass eine ausführliche Labordiagnostik mit Blutausstrich und weiterführender TMA-Diagnostik bei Patienten mit einem systemischen Lupus erythematodes, die sich mit einer Thrombopenie und Anämie – auch im Verlauf unter Therapie – präsentieren, indiziert ist und bei auf Standardtherapie refraktären Verläufen einer TMA hier Eculizumab als eine sog. „Off-label-Therapie“ erwogen werden kann.

## Fazit für die Praxis

Eine ausführliche Labordiagnostik mit Blutausstrich zur weiterführenden Diagnostik einer thrombotischen Mikroangiopathie ist bei Patienten mit einem systemischen Lupus erythematodes, die sich mit einer Thrombopenie und Anämie – auch im Verlauf unter Therapie – präsentieren, indiziert.
